# Estimating the treatment effect in patients with gastric cancer in the presence of noncompliance 

**Published:** 2021

**Authors:** Malihe Safari, Hossein Mahjub, Habib Esmaeili, Sanambar Sadighi

**Affiliations:** 1 *Department of Biostatistics, School of Public Health, Hamadan University of Medical Sciences, Hamadan, Iran*; 2 *Research Center for Health Sciences, Department of Biostatistics, Faculty of Public Health, Hamadan University of Medical Sciences, Hamadan, Iran*; 3 *Principal Statistician and Director of disclosure and posting, Staburo GmbH, Munich, Germany*; 4 *Cancer Research Center of Cancer Institute of Iran, Internal Medicine Group, Tehran University of Medical Sciences, Tehran, Iran *

**Keywords:** Treatment effect, Noncompliance, Time-dependent covariate, Inverse probability of censoring weights, Structural nested model.

## Abstract

**Aim:**

In this study, these methods were used to estimate the treatment effect in patients with gastric cancer in the presence of noncompliance.

**Background:**

In medical sciences, simple and advanced methods are used to estimate treatment effects in the presence of noncompliance.

**Methods:**

This historical cohort study surveyed 178 patients with gastric cancer underwent chemotherapy alone (chemotherapy alone group) and 193 patients underwent surgery and chemotherapy (surgery plus chemotherapy group) from 2003 to 2007 at the Cancer Institute of Imam Khomeini Hospital (Tehran). Demographic and clinical characteristics were extracted from patients' hospital records. The survival of patients was calculated as being from diagnosis to death or to the end of the study. The treatment effect was estimated using three methods: treatment as a time-dependent covariate, IPCW, and Structural Nested Models using STATA and R software.

**Results:**

Fifty-six patients (31.5%) who underwent chemotherapy and 69 patients (35.8%) who underwent surgery and chemotherapy died by the end of the study. The hazard ratio in group I compared to group II was estimated between 1.5 to 2.07 times based on the simple analysis method. The modified hazard ratio was estimated to be 1.21 (95% CI: 1.11-1.32) based on the SNM method. Surgery plus chemotherapy is superior to chemotherapy alone, and it improves the overall survival (OS) rate of gastric cancer patients.

**Conclusion:**

Survival was improved in patients undergoing chemotherapy and surgery together compared to those undergoing chemotherapy alone. The results of the current study suggest that treatment effect can be estimated unbiasedly using the appropriate method.

## Introduction

 Based on GLOBOCAN 2018 data, stomach cancer is the fifth most common neoplasm and the third leading cause of cancer mortality in 2018. About 1 in 12 of all oncological deaths are attributable to gastric cancer, and over a million new cases of gastric cancer are diagnosed worldwide each year (1). Gastric cancer is one of the most common cancers among Iranian men (2).

In most survival studies, randomized clinical trials (RCT) are designed to investigate the treatment effect (TE) in which patients are randomly allocated to treatment and standard groups. Some patients may switch from their allocated group to the other group. This problem is known as noncompliance. Patients often switch from the standard group to the treatment group because of disease progression or clinician decision. Noncompliance is common among patients, and it cannot be prevented due to ethical issues. In such cases, the estimation of TE will be biased and should be adjusted using appropriate methods (3, 4).

In survival studies, various methods have been proposed to adjust TE in the presence of noncompliance. One standard simple method to determine TE in presence noncompliance is the intent-to-treat (ITT) analysis. This method ignores the switching of patients and may provide biased estimates and underestimation of the real effect of treatment. A second simple method is the per-protocol (PP) method which excludes or censors treatment switchers. This method might produce heavily biased results (5-7).

Other simple methods are treated as a time-dependent covariate. This method considers treatment as a time-varying covariate, and it estimates TE by including the time-varying component in a regression model, such as the Cox proportional hazards model. The interpretation of TE is more advanced in this method than in other simple methods, and its results are often biased in the absence of critical confounding factors (8). 

The first advanced method that can be used with observational data sets is inverse-probability-of-censoring weighting (IPCW). In this method, switchers are censored at "time point of cross-over", but patients are weighted according to their probability to switch treatment. All aforementioned methods have both pros and cons in estimating and correcting the TE in presence of switching. IPCW provides an unbiased estimate of TE given that all baseline and time dependent covariates are correctly specified (9,10). 

Structural Nested Models (SNM) comprise another advanced method for estimating causal TE in time-to-event data analysis that has been presented in observational studies in the presence of time-dependent covariates (11). Although TE, based on simple methods, tends to be biased, the SNM method provides semi-parametric stable estimators of causal effect by applying g-estimators. Thus, the SNM method can be used to estimate TE in observational studies like RCT studies. As observational studies are generally conducted on a more extended period and a larger sample size than RCT studies, they usually use more data than RCTs to adjust TE. The SNM method has been much used and well supported by research (11, 12). Many studies in Iran have evaluated TE on patient survival, using conventional models to evaluate TE in the presence of covariates. Nevertheless, the effect of noncompliance has not been included in these studies (13, 14), which may have led to bias in them. Moreover, all previous studies have estimated TE without considering the effects of additional treatment. 

In some survival studies, because of disease progression, medical decision-making, or other reasons, some patients (in this study, patients undergoing surgery group) may switch from their treatment to other supplementary treatment (chemotherapy). The simple methods do not consider this issue, and thus, the effect of treatment will be biased. Therefore, the current study purposed to estimate TE in the presence of noncompliance in patients with gastric cancer using the structural nested model (SNM). 

## Methods

**Patient characteristics and data collection**

This study was conducted as a historical cohort study on 371 patients with gastric cancer in the Institute of Cancer of Imam Khomeini Hospital during 2001-2010. A total of 178 patients underwent chemotherapy (named the chemotherapy treatment group) and surgical treatment was undergone by 193 patients, some of whom switched to chemotherapy treatment (named the surgery plus chemotherapy group). All patient demographic and clinical data was extracted from patient records and included gender (male, female), age at diagnosis in year (<50, 50-60, >60), tumor location (cardia, body, antrum), metastasis (yes, no), relapse (yes, no), stage (2, 3, 4), treatments (surgery, chemotherapy), and hemoglobin (<=11.5, >11.5). To follow the survival status of patients, phone contact with the relatives of patients and patients' referral to the hospital were the follow-up methods of this research. The data of patients in cases of impossible contact and live patients were considered as censored observations. Survival of patients was calculated from diagnosis to death or to end of the study by month. Sixty-five patients received chemotherapy after surgery and were considered switched patients. All patients who received radiotherapy as complementary therapy were excluded from the study. The specific ethics ID code of this study was IR.UMSHA.REC.1397.835.

**Statistical analyses**

The primary purpose of this study was to estimate TE, which was determined by simple and advance adjustment methods such as discussed below.

**Treatment as a time-dependent covariate (simple method)**

In this approach, patients are analyzed according to the treatment they received. This method uses a Cox proportional hazards model in which a binary time-dependent covariate indicates time-periods in which treatment is received [15]. 

**Inverse Probability of Censoring Weights (advanced method)**

ITT analyses in oncology studies when there is subsequent therapy tend to underestimate TE on overall survival. The Inverse Probability of Censoring Weighting (IPCW) is an alternative method, which attempts to reduce the bias caused by treatment change where any patient switches to the alternative treatment. IPCW consists of two steps. In the first step, all baseline time-independent and time-dependent covariates were taken into account using multivariate logistic regression. In the second step, these time-dependent weights were incorporated in a proportional hazards model. To adjust for this bias, the IPCW approach increases the estimation of treatment effect on overall survival (OS) compared to the original ITT analysis. Employing this type of approach may result in more accurate cost-effectiveness results (10, 16).

**Structural Nested Models (SNM) (advance**d **method)**

Structural nested failure time models (SNMs) are causal models which estimate the effect of a treatment on a time-to-event outcome. The SNM was developed for observational datasets. Counterfactual survival times – the survival times that would have been observed if no treatment had been given – are fundamental to SNM methodology. This method uses an accelerated failure time (AFT) model to estimate TE, and it is assumed that exposure to treatment accelerates the time to the event by a factor exp(-ψ). This requires that all factors that contribute to the process that determines whether a patient switches treatment be measured. SNM is applied to estimate counterfactual survival times for a range of possible TEs. Then G-estimation is used to determine a value ψ for TE at each time-point which is independent of counterfactual survival (11-12, 17).

Data was analyzed by STATA v. 11 (StataCorp LP, Texas, USA) and R v. 3.6.1 software. Mean (SD), median, and percentage were used to data. The log-rank test was used to compare the median survival time. Treatment was a time-dependent covariate, and IPCW and SNM methods with and without taking into account covariates using parametric and nonparametric survival models were applied to estimate TE in the presence of noncompliance. A *p*-value of less than 0.05 was considered statistically significant.

## Results

The mean (SD) age at diagnosis in the chemotherapy group and surgical plus chemotherapy group was 60 (12.6) and 58.2 (11.9) years, respectively. Fifty-six patients (31.5%) in the chemotherapy group and 69 patients (35.8%) in the surgical plus chemotherapy group died by the end of the study. The mean and median survival time of patients in the chemotherapy group were 20.9 and 18 months (range: 1-58), respectively, and their one-, three-, and five-year survival rates were 62%, 17%, and 10%, respectively. The mean and median survival in the surgical plus chemotherapy group were 33.8 and 27 months (range: 3-65), respectively, and their one-, three-, and five-year survival rates were 84%, 38%, and 24%, respectively. Table 1 shows the descriptive characteristics of patients.

Subsequently, the effect of variables on survival was investigated in each group, and the results are presented in Table 2. Patient age at diagnosis and differentiation of tumor had significant effects on the survival of patients in the chemotherapy group; gender, metastasis, stage of disease, and hemoglobin significantly affected the survival of patients in the surgical plus chemotherapy group.

**Estimation of TE in simple methods using parametric and semi-parametric models in the absence of covariates**

TE was estimated by a simple method using different semi-parametric and parametric survival models. Based on the goodness-of-fit criterion in Table 3 in both analysis methods, the gamma, lognormal, and log-logistic models were fitted better than other models. According to these models, the time ratio of patients in group I compared to group II was about 0.54-0.66. In all models, the effect of treatment was statistically significant. 

**Table 1:** Demographic and clinical characteristics of gastric cancer patients by treatment group

Variable	Level	Chemotherapy group (group I) n (%)	Surgical group (group II) n (%)
Gender	male	139 (78.1)	140 (72.5)
	female	39 (21.9)	53 (27.5)
Diagnosis age (year)	<50	38 (21.3)	55 (28.5)
	60-50	38 (21.3)	38 (19.7)
	>60	102 (53.3)	100 (51.8)
Differentiation of tumor	well	9 (6.2)	24 (13.4)
	moderate	18 (12.3)	78 (43.6)
	poor	22 (15.1)	55 (30.7)
	unknown	97 (66.4)	22 (12.3)
Tumor location	cardia	55 (40.1)	53 (30.8)
	body	34 (24.8)	49 (28)
	antrum	32 (23.4)	59 (33.7)
	unknown	16 (11.7)	14 (8)
Metastasis	no	51 (28.7)	146 (75.6)
	yes	127 (71.3)	47 (24.4)
Stage	II	0 (0)	39 (20.2)
	III	17 (9.6)	82 (42.5)
	IV	161 (90.4)	72 (37.3)
Relapse	no	163 (91.6)	142 (73.6)
	yes	15 (8.4)	51 (26.4)
Status	death	56 (31.5)	69 (35.8)
	alive	122 (68.5)	124 (64.2)
Hemoglobin	<=11.5	71 (40.6)	33 (17.2)
	>11.5	104 (59.4)	159 (82.8)

**Table 2:** Comparison of median survival rates in gastric cancer patients in each treatment group

Variable	Level	Chemotherapy group		Surgical group	
		Median survival (month)	Log-rank (*p*-value)	Median survival (month)	Log-rank (*p*-value)
Gender	male	15	(0.234)1.4	25	(0.013)6.14
	female	27		40	
Age at diagnosis	<50	14	5.85 (0.041)	30	1.76 (0.416)
	60-50	24		37	
	>60	15		24	
Differentiation of tumor	well	27	7.95 (0.034)	27	5.27 (0.153)
	moderate	12		32	
	poor	-		21	
	unknown	19		-	
Tumor location	cardia	19	2.74 (0.433)	28	1.4 (0.703)
	body	18		32	
	antrum	14		27	
	unknown	10		21	
Metastasis	no	19	0.5 (0.502)	30	4.3 (0.038)
	yes	17		25	
Stage	II	-		-	
	III	12	0.2 (0.765)	26	9.5 (0.008)
	IV	18		20	
Relapse	yes	15	1.8 (0.179)	21	0.3 (0.589)
	no	24		28	
Hemoglobin	<=11.5	17	0.6 (0.472)	23 32	3.6 (0.041)
	>11.5	19			

**Estimating the TE with covariates taken into account**

**Table 3:** Results of model fitting by simple models of estimating TE in the presence of noncompliance using different regression methods without covariates

Analysis method	Model	HR (95% CI)	Time Ratio (95% CI)	Log-likelihood
Treatment as a time-dependent covariate	Cox	1.58 (1.1-2.37)	--	609
	Gamma	--	0.62 (0.47-0.83)	265
	Weibull	1.61 (1.12-2.31)	--	273
	Exponential	1.57 (1.1-2.25)	--	283
	Gompertz	1.6 (1.11-2.13)	--	282
	Log Logistic	--	0.66 (0.51-0.85)	266
	Log normal	--	0.63 (0.47-0.82)	266
IPCW	Cox	2.11 (1.4-3.14)	--	603
	Gamma	--	0.54 (0.41-0.71)	261
	Weibull	1.6-3.36))2.32	--	268
	Exponential	1.74 (1.22-2.5)	--	282
	Gompertz	2.01 (1.39-2.9)	--	278
	Log Logistic	--	0.58 (0.45-0.74)	262
	Log normal	--	0.54 (0.42-0.71)	261

IPCW: Inverse-probability-of-censoring weighting; CI: confidence interval

**Table 4:** Results of model fitting by simple models of estimating TE in the presence of noncompliance using different regression methods and taking covariates into account

Analysis method	Model	HR (95% CI)	Time Ratio (95% CI)	Log-likelihood
Treatment as a time-dependent covariate	Cox	2.07 (1.12-3.82)		243
	Gamma		0.58 (0.38-0.88)	119
	Lognormal		0.58 (0.37-0.91)	119.7
	Log- logistic		0.61 (0.41-0.95)	121
IPCW	Cox	1.61 (0.78-2.2)	--	244
	Gamma		0.62 (0.37-1.02)	120
	Lognormal		0.67 (0.41-1.12)	121
	Log- logistic		0.72 (0.44-1.23)	123

IPCW: Inverse-probability-of-censoring weighting; CI: confidence interval

**Figure 1:**
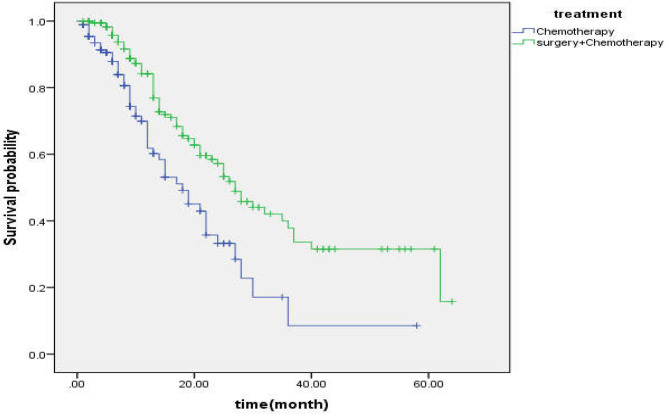
Survival of gastric cancer patients by treatment group

The adjusted TE was estimated in the presence of covariates. The results presented in Table 4 show that the gamma and lognormal models in both analysis methods were the best fitting. With treatment as a time-dependent covariate, all models detected TE as being statistically significant; with the IPCW method, however, TE was not significant.

**Estimation of TE using SNM as an advanced method with and without considering covariates**

Finally, TE was estimated (95% CI) using the g-estimation method without considering the covariates, and the hazard ratio (95% CI) was equal to 1.14 (1.09-1.38); that is, individuals in Group I are at risk of dying at a rate of approximately 1.12 times higher than those in Group II. The adjusted TE with covariates taken into account was also estimated, and the modified hazard ratio was equal to 1.21 (1.11-1.32). Based on the SNM method, the patients in Group I are at a 1.22-times greater risk of death than patients in Group II.

Figure 1 shows the survival of gastric cancer patients by treatment group.

## Discussion

This research used several methods to estimate TE in the presence of noncompliance. The use of simple methods usually delivered biased results. As the results showed, the accuracy and validity of complex methods reduce and sometimes exclude bias in TE estimation when there are confounding variables and appropriate sample sizes in observational and clinical trial studies. In such cases, patients are followed up until switching time. Similar to the current study, bias and poor performance in dealing with noncompliance have been reported in estimating TE (3-4; 18-21).

Five-year survival rates in this study were 24% among patients in the surgery plus chemotherapy group and 12% among those in the chemotherapy group. Consistent with this study, Shi et al. reported 5-year OS rates of 25% in the chemotherapy plus surgery group and 10% in the chemotherapy alone group (22). Li et al., however, estimated the 5-year OS rate to be about 10% in the chemotherapy plus surgery group (23).

The median OS rates in the current study were 18 months for patients undergoing chemotherapy and 27 months for patients undergoing chemotherapy plus surgery, and the difference between the groups was significant (*p*<0.05). Park showed that the median of OS for patients who received conversion surgery and those who received chemotherapy only was 43.6 months and 14.0 months, respectively (24). 

Cho et al. reported median survival rates in the surgery plus chemotherapy group compared to the chemotherapy alone group to be 19% and 9%, respectively (*p*<0.001) for patients with advanced gastric cancer (25). Similar to the current study, the median OS in a study by Yuan et al. was 23.6 months for the gastrectomy group and 13.8 months in the non-gastrectomy group (26). In Shi et al., the median OS was 15.9 months for patients who underwent chemotherapy plus surgery and 10.9 months for patients treated with chemotherapy alone (22).

The difference between these studies may be a concern regarding different clinical characteristics and quality of treatment. 

Based on all hazard-based models in the presence and absence of covariates, the present study determined the hazard of death in patients undergoing chemotherapy compared to patients undergoing surgery plus chemotherapy to be about 1.5 to 2.5 times greater. Based on the time ratio model, the results showed that the duration of survival in patients who underwent chemotherapy was estimated to be about 0.54 to 0.66 times that of patients who underwent surgery plus chemotherapy. According to the SNM method, the chemotherapy group compared with the surgery plus chemotherapy group had a significantly lower likelihood of overall survival (adjusted hazard ratio for death was 1.21 (95% CI, 1.11 to 1.32; *p* < 0.05). The results of observational cohort studies showed that estimated hazard of death in patients with local recurrence and distant metastasis undergoing chemotherapy to be between 1.3 and 1.84 (14, 27-29). In their meta-analysis study, Wu et al. showed that the HR of death in the chemotherapy alone group compared to the surgery plus chemotherapy group was 2.33 (95% CI=1.54-3.45) (30). Cho et al. assessed TE in patients with advanced gastric cancer. Their results showed that after adjusting for covariates in multivariate analysis, the HR for death in the chemotherapy group was 2.18 (95% CI: 1.37 to 3.48) (25). After adjusting by the propensity score, which is used to reduce selection bias by equating groups based on baseline covariates in observational studies, Yuan et al. concluded that the HR of death in the non-gastrectomy group was 2.38 (1.21-4.76) (26). Shi et al. demonstrated that patients who underwent surgery plus chemotherapy had prolonged overall survival compared to those undergoing chemotherapy alone (HR = 0.61, 95% CI: 0.51‐0.73, *p* < .001) (22). The clinical and demographical characteristics of patients and treatment agents, especially in the chemotherapy alone group, may differ among all implemented studies; nevertheless, consistent with the current study, the results confirmed the superiority of surgery plus chemotherapy. The current results regarding the hazard ratio of death in the chemotherapy group compared to the surgery plus chemotherapy group estimated using the SNM method did not differ greatly from those of other studies. This similarity between the current results and those of the aforementioned observational and clinical trials may be due to the conditions of the patients under study (in terms of clinical features, different treatment regimens, and patient conditions) and the study design used to estimate TE (clinical trial or observational study). Some studies have investigated patients with specific conditions. For example, some studies were conducted on patients in advanced stages of the disease; in others, all patients had local recurrence or distant metastasis. These conditions can affect the hazard of death for patients.

The results of this study showed that gamma, lognormal, and log-logistic models were better fitted to the data. Similar to the present study, the results of most studies have shown that the Weibull model was the best parametric model for estimating TE in cancer studies (31-34). The reason for the difference between the appropriate model in the present study and that of other studies may be the study design (clinical trial or observational), because randomization in clinical trials eliminates the effect of all confounders that may influence TE. Furthermore, the switching time in experimental studies is recorded precisely. In contrast, in observational studies, some patients who switch to supplementary treatment are missed, and their information is not included in the analysis. This practice may lead to bias in the results.

It is noteworthy that data in the present study was collected from an observational study, and the SNM method can be used to adjust the estimation of TE on overall survival in the presence of noncompliance in observational studies. The SNM model with and without covariates estimated lower hazard ratios than other methods and had higher accuracy (narrow confidence interval). The results of simulation studies have shown that the SNM method performs much better for observational data than simple methods if the noncompliance rate is low. This method also works better than simple methods to adjust noncompliance in TE, even for high censoring and noncompliance rates (3, 15, 34). 

**Study weaknesses and strengths**

Because the current study design was a retrospective cohort study that included all patients referred to Tehran Cancer Institute in Iran, some variables had missing data, especially information about the differentiation of tumor and tumor location. Exact dates of gastric surgery and chemotherapy were unavailable in some patients' records. Moreover, although information regarding the receipt of chemotherapy was available, there was no data regarding the number of cycles or which chemotherapy agents were administered. This could lead to a survival bias. Finally, a selection bias may be present in the current data, considering that this study was conducted in a single center. 

The main strength of the current study is the use of an advanced method for TE estimation in gastric cancer patients. 

Further research is still needed, but the results of the present study suggest that surgery plus chemotherapy is superior to chemotherapy alone, and it improves the OS of gastric cancer patients. Although various statistical methods are available for adjusting estimates of TE for noncompliance, most are not suitable for use. Methods based on g-estimation appear to be more appropriate for estimating effectiveness in the presence of real-world adherence. Furthermore, in observational studies, all primary and time-dependent covariates should be recorded so as to use appropriate methods to adjust TE. In conclusion, a more well-designed and high-quality study is needed to validate the significance and efficacy of treatment effect for gastric cancer study.
